# Community-Oriented Dentistry Education: A Narrative Review

**DOI:** 10.7759/cureus.76986

**Published:** 2025-01-06

**Authors:** Bhavna Jha Kukreja, Pankaj Kukreja

**Affiliations:** 1 Periodontology, Department of Preventive Dental Sciences, College of Dentistry, Gulf Medical University, Ajman, ARE; 2 Oral and maxillofacial surgery, Department of Biomedical and Dental Sciences, Faculty of Dentistry, Al-Baha University, Al Aqiq Campus, Al Baha, SAU

**Keywords:** community-oriented curriculum, dentistry, health profession’s education, oral health, telehealth

## Abstract

Community-oriented dental education (CODE) is a teaching and learning approach in dentistry that occurs at a location outside the traditional dental education institution which bridges the gap between theoretical knowledge and practical application. CODE programs differ in terms of student involvement, rotation duration, types of services offered, and the structure of community partnerships. The nature and structure of the programs that are implemented in specific places determine the precise impacts of the CODE. Hence, the current narrative review highlights the determinants of CODE, challenges associated with the implementation of CODE, and opportunities for advancement. The six determinants highlighted consist of addressing community needs, promoting social responsibility, implementing CODE for oral health challenges, enhancing cultural competence and sensitivity, collaborating with organizations, and integrating public health principles. Moreover, it illustrates opportunities for advancement consisting of technological innovations, global exchange programs, and interdisciplinary collaboration which enhance the effectiveness and impact of CODE. However, the review discusses various challenges that involve resistance to change, limited resources, and difficulties in assessing outcomes that result in the complexities of the implementation of CODE. Hence, the CODE bridges knowledge and applied education models, allowing for a comprehensive approach to training graduates to tackle oral health inequalities and advance equity.

## Introduction and background

A community-oriented curriculum (COC) is defined as an educational framework tailored to address the specific needs, interests, and concerns of the community in which it is implemented [1]. Numerous calls have been made for significant reforms in health professionals’ education to more effectively address the health needs of populations and communities [2]. This necessitates a shift in education toward community-focused learning, ensuring healthcare professionals are adequately prepared to work with diverse populations and communities.

As a result, community-oriented education programs, recognized as an innovative teaching approach, are increasingly regarded as essential. These programs provide students with the opportunity to gain knowledge in real-world settings, thereby enhancing meaningful changes in attitudes and perspectives [2,3]. It can facilitate students in developing a more comprehensive understanding and recognition of the broader social, economic, and cultural determinants of health, as well as their influence on individual health outcomes and healthcare delivery [4,5]. Community-oriented experience allows the application of students’ professional and clinical skills into real-world settings, offering them a deeper and more comprehensive understanding of their patients across various social contexts beyond what typical institution clinical encounters can provide [2]. This prepares students to understand the needs of their future patients, enabling them to provide care at all levels within the healthcare settings [6,7].

Community-oriented dental education (CODE) is a teaching and learning approach in dentistry that occurs at a location outside the traditional dental education institution [8]. The CODE educational approach seeks to bridge the gap between theoretical knowledge and practical skills, overall developing dental professionals who are clinically competent, empathetic, and community-oriented [8].

CODE programs are usually collaborative efforts between dental institutions, community clinics, public health services, private dental practices, and public hospitals, leading to a diverse and comprehensive learning environment [8]. Dental students in these environments actively engage in patient care, diagnosis, treatment planning, and clinical procedures, all under the supervision of experienced dental practitioners. This hands-on learning approach fosters critical thinking, enhances problem-solving skills, and builds clinical confidence [8].

CODE programs differ in terms of student involvement, rotation duration, types of services offered, and the structure of community partnerships [8]. The nature and structure of the programs that are implemented in specific places determine the precise impacts of the CODE, e.g., in contrast to observation-based rotations, models where students give dental care in community settings under supervision offer invaluable practical clinical experience [8]. Additionally, some CODE programs offer opportunities for students to integrate their experiences in the community. This reflection enables students to evaluate their clinical experiences, recognize their strengths and weaknesses, and determine areas for improvement, allowing a deeper level of self-directed learning [9].

Although considerable research has been conducted on this topic, there has yet to be a comprehensive review of the outcomes and objectives of integrating community education into the dental curriculum. Hence, this narrative review aims to synthesize determinants of CODE, providing insights into curriculum development, challenges encountered, and opportunities for enhancing educational outcomes.

## Review

The Scale for the Assessment of Narrative Review Articles (SANRA) was utilized to search the articles, formulate inclusion and exclusion criteria, critically appraise the literature, and appropriately present the evidence [10]. A comprehensive literature search was performed on Science Direct, PubMed, and Google Scholar databases from 2019 to 2024. The keywords along with the Boolean operators utilized consisted of, “Dental Education”, OR “Dentistry Education”, AND “Community-based Teaching”, OR “Community-based Education” to inculcate appropriate literature. The inclusion criteria for screening were studies focused on community education within the field of dentistry, including program evaluations, accounts of curricular innovations, conceptual articles, editorials, published reports, and peer-reviewed studies of all study designs. Studies were selected from various countries to offer a comprehensive view of global research on community education in dentistry, published between 2019 and 2024, in English, and with full-text availability. However, articles that discussed community-based programs outside the scope of dentistry did not focus on education, articles with only a title and no abstract, studies not published in English or lacking an available English translation, those with unavailable full text, and those providing insufficient contextual information were excluded.

Two independent reviewers evaluated the articles to determine their suitability for inclusion in the review. Initially, titles and abstracts were screened to remove duplicates. Next, the selected articles were re-screened to exclude those that did not meet the eligibility criteria. Finally, the remaining articles were assessed based on their full text to confirm eligibility. Any discrepancies or disagreements between the reviewers were resolved through discussion and consensus. The critical narrative approach was used to synthesize the results of the included studies [11]. The studies utilized various research methodologies and outcome measures, resulting in a considerable level of heterogeneity.

Determinants of community-oriented dental education (CODE)

Aligning with Community Needs

Tailoring education to specific health issues is central to CODE. Needs assessments through surveys, focus groups, and stakeholder interviews help identify priorities like oral hygiene education in underserved areas, access to care, and preventive strategies that guide curriculum development to address local challenges [1]. Tailored education fosters community trust and enhances program impact. Community surveys and focus groups identify pressing issues such as untreated dental caries, periodontal disease, and access barriers in underserved populations whereas, stakeholder engagement involves collaborating with local health authorities, patients, and advocacy groups which ensures that curriculum addresses priority concerns like preventive care and health promotion, and employs objective-oriented modular systems [1,12,13].

Community-Oriented Curriculum for Addressing Oral Health Issues

This focuses on aligning dental education with the most pressing oral health issues in the community [1]. These problems are identified through epidemiological studies, needs assessments, and direct engagement with target populations [1]. The key problems encountered consist of dental caries (tooth decay), periodontal (gum) diseases, edentulism (total tooth loss), oro-dental trauma, limited access to dental care in rural and underserved areas, oral cancer, tobacco-related diseases, and oral health inequalities [13,14]. The CODE implications mainly concentrate on introducing community-based preventive programs, such as fluoride varnish applications and dental sealants, including epidemiology modules focusing on caries risk factors and population-level interventions, emphasizing early detection through community health screenings, and teaching public health strategies for improving access to periodontal care [13,14].

Engaging members of the community in the development of the curriculum enables educational programs to remain responsive and relevant [2]. Engagement may involve participatory research projects, clinical rotations, and health promotion initiatives. These collaborations empower communities while enriching students' educational experiences [8,15]. Service-learning opportunities enable students to engage in projects, promoting practical learning and building trust among the population. Moreover, addressing real-world problems in underserved communities helps students develop practical skills while allowing populations to tackle their oral health challenges. Communities work alongside dental institutions to design and implement health interventions, leading to shared ownership [16]. Furthermore, incorporating rotations in rural health clinics to expose students to resource-limited environments, training students in mobile dentistry and telehealth solutions, integrating modules on counseling and preventive strategies for tobacco cessation, teaching students to perform oral cancer screenings during community visits, engaging students in service-learning projects to address these disparities and embedding discussions of health equity and advocacy should be incorporated in the dental curriculum [13,14].

Cultural Competence and Sensitivity

For equipping dental students to serve diverse populations effectively, training in cultural competence is essential [17,18]. Experiences like community fieldwork, language courses, and cultural programs that are immersive enhance students’ understanding of patients' sociocultural contexts [19]. It improves adherence to interventions and strengthens relationships between the dentist and the patient. Additionally, communication skills and language training can help overcome restrictions related to language, while encouraging students to reflect on their assumptions and biases about different cultural contexts improving their ability to deliver equitable care. Furthermore, to expand dental students' understanding of global health disparities, institutions can integrate cross-cultural exchange programs [19,20].

Social Accountability

CODE focuses on aligning dental education with the needs of society. The frameworks such as the social accountability grid assess how well the curriculum responds to the priorities of the community. Collaborations with organizations and local health authorities are essential for effective dental education [21,22]. CODE encourages dental graduates to perceive dentistry as a profession driven by service. Additionally, student involvement in advocacy programs encourages them to facilitate oral health equity, tackling disparities in care access and affordability. Moreover, dental graduates should uphold principles of equity and fairness in their practice, focusing on populations that are mainly underserved [1,23].

Integration of Public Health Principles

In many countries, CODE is an integral part of dental education, aiming to address oral health issues by promoting preventive and community-focused care. However, gaps remain in regions where CODE is not included in the curriculum such as in many developing countries as they face challenges due to limited resources, faculty shortages, and inadequate infrastructure, which can hinder the inclusion of comprehensive CODE programs. This highlights the need to prioritize the integration of such courses globally to equip dental professionals with the skills to address diverse population needs effectively by overcoming these challenges.

Incorporating courses associated with public health promotes a deeper understanding of oral health in societal contexts, preparing students for population-oriented approaches and preventive care [1,24]. Epidemiology courses primarily focus on teaching students about the incidence and risk factors of oral diseases, consequently preparing them to overcome systemic challenges should be implemented [1]. Moreover, to reduce the burden of preventable diseases, preventive care modules provide students with the knowledge to design and implement oral health programs [23]. Furthermore, an interdisciplinary approach facilitates collaboration with public health professionals, introducing students to integrated care models [24]. Hence, public health integration ensures that graduates view oral health as an essential component of overall well-being.

Collaboration with Organizations

Partnerships with government and non-governmental organizations enrich community-based learning by providing practical exposure to health challenges and enhancing advocacy skills while fostering community-related solutions [1,23]. Partnerships provide financial and logistical support for community-oriented projects [25]. Additionally, internships and service-learning projects with organizational partners offer real-world insights into oral health challenges. Students learn to influence oral health policy through collaborative initiatives with NGOs [1,12,13]. Hence, collaboration ensures that CODE is not only effective but also sustainable.

Challenges in implementing CODE

Implementing a community-oriented framework in dental education faces several barriers, primarily due to the systemic, cultural, and logistical shifts it requires. The most commonly encountered challenges are described as follows:

Resistance to Change in Educational Systems

Many dental institutions still adhere to traditional, clinically-focused curricula, emphasizing technical skills over social accountability and public health. Faculty and administrators may resist adopting new methodologies, perceiving community-oriented approaches as less rigorous or outside the scope of clinical dentistry [1]. For instance, faculty with limited exposure to community-oriented practices may lack the confidence or expertise to teach these aspects effectively. Additionally, institutions might prioritize technical competencies in response to accreditation pressures, sidelining CODE initiatives [1,8].

Resource Limitations

CODE implementation requires significant investments in infrastructure, faculty training, and partnerships with community organizations [2,8]. Resource-limited institutions, particularly in developing countries, may struggle to allocate budgets for these initiatives. E.g., insufficient funding for community rotations, mobile clinics, or telehealth services limits students' opportunities to interact with underserved populations and is associated with high costs of integrating public health courses or cultural sensitivity training into the existing curriculum [8,26].

Faculty Development Gaps

Many educators lack training in public health, cultural competence, or community engagement, which are core components of CODE. Faculty development programs are often underfunded or non-existent, limiting their ability to mentor students in community-oriented practices, e.g., lack of workshops or certifications on interdisciplinary and community-based teaching methods or overburdened faculty may view additional CODE responsibilities as unmanageable [1,8].

Balancing Clinical and Community Learning

Traditional dental education emphasizes individual patient care and clinical proficiency, often at the expense of community health perspectives [3]. Integrating community-oriented learning into a packed curriculum can be challenging without compromising other core competencies. For instance, scheduling conflicts between clinical rotations and difficulty assessing community engagement outcomes alongside clinical milestones [26].

Measuring Impact and Outcomes

It is challenging to evaluate the effectiveness of CODE due to its diverse and wide-ranging objectives, including enhancing cultural competence, public health literacy, and health equity [2] for which institutions frequently lack standardized frameworks or metrics to assess these outcomes. E.g. challenges include limited data on the long-term effects of CODE learning on the outcomes as well as quantifying improvements in social accountability or cultural sensitivity among students [26].

Limited Community Partnerships

Constant efforts and resources are required to establish and sustain meaningful partnerships with community organizations [1,8]. However, due to negative experiences encountered previously, mistrust, or due to lack of reciprocity, communities may be reluctant to engage with dental institutions. E.g. Due to a lack of frameworks to ensure mutual benefit for both students and community stakeholders and due to logistical and geographical barriers, dental institutions may face challenges in establishing partnerships with rural communities [1,26].

Opportunities for advancement in CODE

The opportunities for advancing CODE align with the evolving needs of communities and educational institutions. For sustained impact, institutions must leverage these opportunities to embed community-oriented principles deeply into their curriculum. The various opportunities are described as follows:

Integration of Emerging Technologies

Telehealth enables remote consultations and education in underserved areas, exposing students to innovative care delivery models while improving access for rural populations. Technologies involving augmented reality (AR) and virtual reality (VR) allow students to practice procedures in simulated environments, enhancing their preparedness for real-world scenarios [1]. For instance, VR modules in community-based settings could simulate interactions with diverse populations. Moreover, smartphone applications designed for oral health education can serve both students and community members as these apps can provide real-time feedback and tutorials on dental hygiene practices, and patient management guidelines, enhancing the learning experience while simultaneously addressing community needs [1]. Artificial intelligence (AI) can be utilized to customize learning for students by identifying their limitations and strengths through adaptive learning platforms. AI-driven data analytics can also predict community-specific oral health trends, helping students and institutions plan targeted interventions [27].

Expansion of Interdisciplinary Education

Collaboration with medical, nursing, and public health students fosters holistic care approaches and improves teamwork in addressing oral health disparities [2,3]. Cross-disciplinary case studies and community projects integrate knowledge of systemic and social determinants of health that interplay with oral health [26]. Additionally, the integration of interdisciplinary learning modules can help prepare students to encounter multifaceted challenges. E.g., modules determining the association between oral health and chronic diseases can prepare students for collaborative care models. Moreover, community projects provide students with hands-on training experience developing communication and promoting teamwork skills [26].

Standardization of Community-Oriented Competency Frameworks

Developing global frameworks for competencies in community-oriented dentistry will unify standards and ensure graduates are adequately prepared [1,2]. Moreover, institutions can benchmark against established models like the World Health Organization’s (WHO) social accountability framework to align with global priorities [26]. Additionally, AI competency frameworks for students should be utilized for CODE [28]. Collaborative efforts among dental schools worldwide can lead to the development of universally recognized competency frameworks [1,2]. This ensures that students are trained to meet the demands of a globalized healthcare landscape. Accrediting bodies can incorporate CODE as a mandatory component in their evaluation criteria. This would encourage institutions to prioritize community engagement and public health principles within their curricula. Establishing mechanisms for the periodic review of competency frameworks ensures that they remain relevant to evolving community needs and advancements in dental science. Feedback from practitioners, educators, and community representatives can guide these updates [1,2].

Globalization and Cultural Exchange Programs

Exchange programs expose students to global health challenges, enabling them to learn best practices from diverse healthcare systems [24]. Cultural immersion initiatives teach students to adapt care delivery to varied sociocultural contexts [19,20], e.g., dental schools partnering with international institutions for short-term exchanges allow students to address oral health challenges in low-resource countries. Moreover, incorporating global health modules into dental education ensures that students are aware of the broader context of oral health issues. Advances in technology enable virtual exchange programs, where students can engage in cross-cultural learning without physical travel. Virtual platforms can facilitate joint seminars, case discussions, and collaborative research projects [24]. Furthermore, exposure to international conferences and workshops allows students to network and learn from global leaders in the field.

Research and Data-Driven Decision-Making

Utilizing community-specific epidemiological data ensures that the curriculum remains relevant and impactful [1,12], e.g., data on the prevalence of dental caries or periodontal disease in a particular community can guide the inclusion of targeted prevention and treatment strategies in the curriculum. Additionally, encouraging student-led research projects in communities generates insights for future interventions leading to innovation and empowerment consequently enhancing critical thinking and enabling them to be proactive contributors [13,29] which leads to an increase in the overall performance of the community as well as the students. Moreover, feedback Loops for continuous Improvement can include tracking the impact of community outreach activities on population health outcomes or evaluating the competency of graduates in addressing real-world challenges. Furthermore, encouraging students and faculty to present their research findings at conferences and publish them in high-impact journals ensures that valuable insights are shared with the wider academic and professional community [29].

Strengths and limitations

The review systematically explores key determinants, such as cultural competence, community engagement, and social accountability, offering a holistic perspective on CODE implementation. It draws insights from a broad range of global studies, ensuring a multicultural and multi-contextual understanding of CODE. The review not only identifies barriers but also offers actionable recommendations for overcoming them, making it practical and progressive. By emphasizing participatory research, service-learning projects, and organizational collaborations, the review bridges knowledge and applied education models. However, the review highlights certain limitations which involve the inclusion of studies with varying methodologies which may limit the ability to generalize findings across different educational settings, and consideration of literature only in English which may have resulted in the exclusion of valuable research published in other languages. Additionally, focusing on articles published between 2019 and 2024 might overlook earlier foundational studies that could provide additional insights into CODE.

## Conclusions

CODE offers a transformative framework for addressing oral health disparities while equipping dental professionals to address the complex needs of diverse populations. By focusing on determinants such as relevance to community needs, cultural competence, and public health integration, CODE connects knowledge with practical implications. Despite challenges like resource limitations and resistance to change, opportunities such as technological advancements and interdisciplinary approaches provide a path for enhancing CODE. A sustained commitment to embedding community-oriented principles in the dental curriculum will be crucial for fostering socially accountable dental professionals and improving oral health equity worldwide.
